# A proof-of-concept study on high-pressure freezing for cryopreservation

**DOI:** 10.1093/pnasnexus/pgag065

**Published:** 2026-03-20

**Authors:** Fang Song, Mayuko Sato, Yuya Toyama, Taiyo Ishikawa, Fumiya Tokito, Takeshi Katsuda, Kiminori Toyooka, Yasuyuki Sakai, Masaki Nishikawa

**Affiliations:** Department of Chemical System Engineering, Graduate School of Engineering, The University of Tokyo, 113-8656 Tokyo, Japan; Center for Sustainable Resource Science, RIKEN, 230-0045 Yokohama, Japan; Department of Chemical System Engineering, Graduate School of Engineering, The University of Tokyo, 113-8656 Tokyo, Japan; Department of Chemical System Engineering, Graduate School of Engineering, The University of Tokyo, 113-8656 Tokyo, Japan; Department of Chemical System Engineering, Graduate School of Engineering, The University of Tokyo, 113-8656 Tokyo, Japan; Department of Chemical System Engineering, Graduate School of Engineering, The University of Tokyo, 113-8656 Tokyo, Japan; Center for Sustainable Resource Science, RIKEN, 230-0045 Yokohama, Japan; Department of Chemical System Engineering, Graduate School of Engineering, The University of Tokyo, 113-8656 Tokyo, Japan; Department of Chemical System Engineering, Graduate School of Engineering, The University of Tokyo, 113-8656 Tokyo, Japan

**Keywords:** cryopreservation, vitrification, high-pressure freezing, cryoprotective agents, fracture

## Abstract

Cryopreservation by vitrification typically requires 30–50 v/v% of cytotoxic penetrable cryoprotective agents (CPAs) to prevent ice crystal formation during freezing and thawing, limiting its broader application. Since pressure suppresses ice crystallization, applying high pressure during vitrification may enable reducing CPA concentrations while maintaining cell viability. In this study, we used a high-pressure freezing (HPF) device, commonly used for cryofixation, to successfully cryopreserve 2D cell monolayers and 3D cell spheroids with 20–30 v/v% penetrable CPA. Compared with commonly used plunge freezing, HPF cell monolayers exhibited higher postthaw viability and better retention on the substrate, allowing for subsequent proliferation. HPF cell spheroids showed improved cell viability, metabolic activity and maintained cell–cell adhesion. Developing HPF devices specifically for cryopreservation, in combination with advanced warming techniques, holds promise for achieving vitrification with low or even no CPA.

Significance Statement Vitrification is widely used for the cryopreservation of cells, tissues, organs, and organisms. However, it typically requires high concentrations of cryoprotective agents (CPAs). Although the potential of high pressure to suppress ice formation has been recognized, its application in cryopreservation remains underexplored. Herein, we adapted a high-pressure freezing device commonly used in cryofixation and demonstrated that vitrification under 210 MPa significantly improved the postthaw viability of cell monolayers and cell spheroids. Cryopreserving cell monolayers and cell spheroids can reduce the time required for cell culture and directly provide ready-to-use cellular formats, which is beneficial for drug metabolism studies and high-throughput screening. In addition, we found that using dextran as a nonpenetrating CPA prevented fracture formation during vitrification.

## Introduction

Vitrification transforms biological samples from a liquid state to a glassy, amorphous state through rapid cooling and high concentrations of cryoprotective agents (CPAs) (1). Unlike conventional slow freezing—which induces extracellular ice formation, leading to cell dehydration and a sharp increase in intracellular CPA concentration (2)—vitrification yields superior outcomes in various biological samples, including reproductive cells (3), stem cells (4), and multicellular 3D structures (5, 6).

In vitrification, a trade-off lies between the CPA's cytotoxicity and its ice-inhibiting ability. Lowering CPA concentration typically requires higher cooling and warming rates to prevent ice crystal formation; however, sample size and thermal conductivity limit these rates. The major challenges in vitrification include low sample viability caused by CPA cytotoxicity and ice crystal damage and limitations in scaling up sample volumes. To address these issues, various strategies have been explored, including enhancing cooling and warming rates (7–10), developing low-toxicity CPA (11–14) and employing physical strategies such as hydrogel encapsulation to mitigate ice crystal formation (15, 16).

Applying pressure can suppress crystallization similarly to solute addition (17, 18). As pressure increases, both the freezing point and homogeneous nucleation temperature of water decrease, reaching their lowest values at 210 MPa, at which the freezing point decreases from 0 to −22 °C, while the homogeneous nucleation temperature decreases from −38 to −92 °C (19). Among cryofixation techniques used in microscopy, high-pressure freezing (HPF) at 210 MPa enables vitrification under low-molarity or CPA-free conditions, allowing preservation of ultrastructural details with minimal structural alterations (20–22). In cryopreservation, a recently developed pressure-based approach, isochoric vitrification, leverages the expansion caused by ice formation within a fixed-volume chamber to increase pressure, thereby suppressing crystallization in the remaining unfrozen fraction (23, 24). Although isochoric vitrification can theoretically generate pressures of up to 210 MPa (i.e. the triple point pressure of water/ice Ih/ice III), current studies typically rely on the kinetic consequence of isochoric confinement to reduce the probability of ice nucleation during cooling and warming, thereby avoiding pressurization (23, 25). Additionally, the thermal contraction of the sample needs to be considered (26, 27). Meanwhile, studies on cryopreservation under externally applied high pressures remain scarce, with only very few reports available. In a pioneering study (28), Fahy et al. vitrified kidney slices under 100 MPa and found that high pressure reduced about 5 v/v% penetrable CPAs to achieve visually ice-free morphology. Additionally, exposure to 100 MPa for several minutes caused minimal tissue damage (28, 29). While their work highlighted the potential of vitrification via HPF, the heat transfer challenges in large sample volumes limited their approach; the conclusions were primarily based on visual assessment of postfreezing morphology, without subsequent thawing and viability evaluation.

Here, we conducted a proof-of-concept study on vitrification via HPF at 210 MPa for cryopreservation. In accordance with common terminology, we use the term HPF throughout this study. Although the term “freezing” is widely used, the primary objective of HPF, both in cryofixation and in our work, is to achieve vitrification and avoid ice crystal formation, which can otherwise lead to damage or artifacts. We used an HPF device commonly employed for cryofixation, the Leica EM ICE. After mounting the carrier onto the specimen holder, vitrification can be achieved with a one-click operation (Fig. S1a); the device completes pressurization via compressed gas and subsequent cooling with a liquid nitrogen jet within 10 ms. Both the pressure and the cooling rate are set by the instrument's default operating parameters (Fig. S1b, Video S1). The cooled samples are then stored in a built-in liquid nitrogen container for retrieval. We optimized CPA formulations for cryoprotection and mechanical damage mitigation. With optimized CPA, we successfully cryopreserved both 2D and 3D biological samples. This study expands current vitrification paradigms by demonstrating pressure as another factor in cryopreservation, offering a new dimension to current strategies.

## Results

### Experimental design and workflow

**Figure 1:**
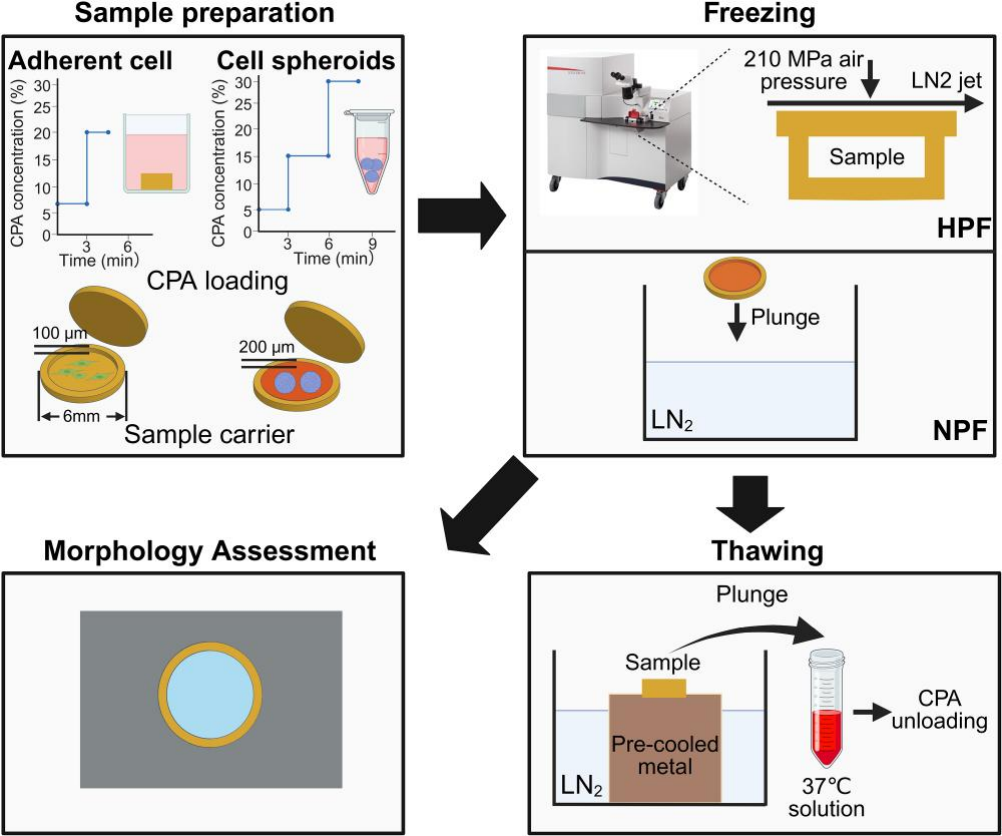
Overview of the study. Prior to freezing, samples underwent CPA loading. Cell monolayers and cell spheroids were loaded into type A carriers: 100 μm deep side of carriers for 2 μL samples and 200 μm deep side for 4 μL samples. Freezing was performed using two methods: HPF and NPF. In HPF, samples were pressurized to 210 MPa, followed by vitrification via a liquid nitrogen (LN_2_) jet. In NPF, samples were directly plunged into liquid nitrogen. Thawing was conducted by first placing frozen samples on a precooled metal surface for 3 min to remove residual liquid nitrogen, then plunging them in a 37 °C solution for CPA unloading. Some frozen samples were kept at liquid nitrogen temperature for morphology assessment.

Experiments were conducted as summarized in Fig. 1 (see Materials and methods for details). Briefly, samples were mounted on copper carriers (Leica). Prior to freezing, CPAs were equilibrated with the samples, and HPF was performed using a Leica EM ICE. For comparison, samples were also plunge frozen directly into liquid nitrogen, representing a conventional approach referred to here as normal pressure freezing (NPF) (7, 30). In accordance with common terminology, we refer to the methods used in this study as HPF and NPF. However, our actual purpose is to vitrify the samples to prevent ice crystal formation. Before thawing, each sample was placed for 3 min on a metal surface precooled with liquid nitrogen to remove residual liquid nitrogen around the sample. This step was crucial in preventing the Leidenfrost effect, where vaporized liquid nitrogen forms a low-conductivity gas layer that hinders heat transfer and can even occasionally suspend the sample above the warm solution (Video S2).

### Effect of CPA composition on freezing morphology

We first tested a commonly used vitrification CPA formulation, ethylene glycol (EG)/dimethyl sulfoxide (DMSO) + trehalose, in HPF experiments. EG and DMSO, as penetrable CPAs, diffuse across the cell membrane and interact with water molecules to suppress intracellular ice formation but are generally more toxic than nonpenetrable CPAs. Trehalose, as nonpenetrable CPA, unable to cross the cell membrane, primarily promotes osmotic dehydration to concentrate intracellular CPAs and has low toxicity. However, cell monolayers cryopreserved with CPAs containing trehalose exhibited severe cell detachment and low postthaw viability. In contrast, replacing trehalose with dextran, a commonly used nonpenetrating CPA in HPF, markedly preserved the cell layer and improved cell viability (Fig. S2).

**Figure 2:**
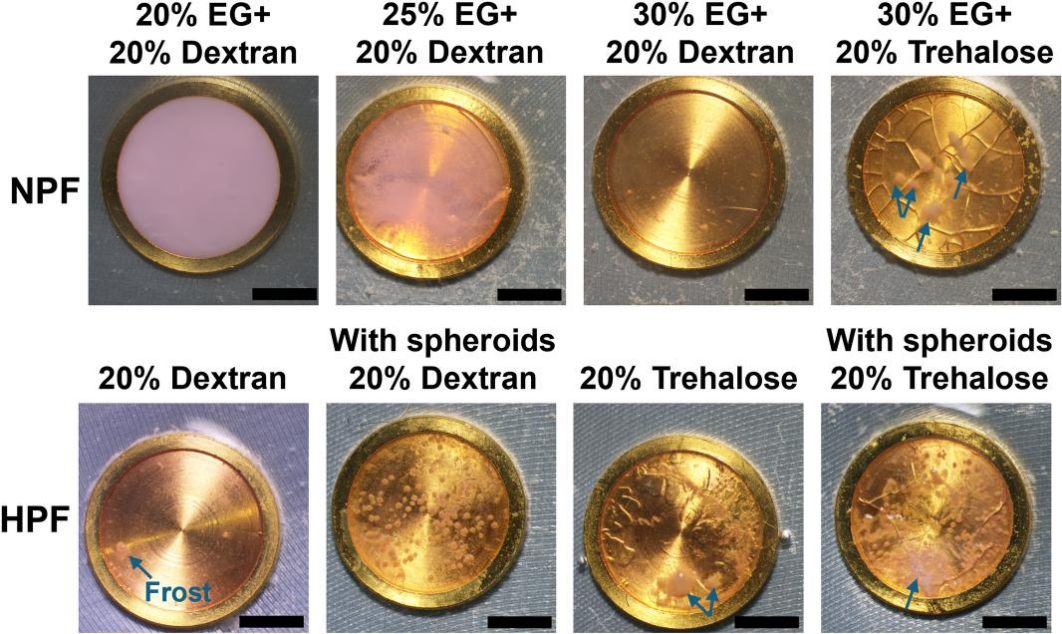
Visual assessment of freezing morphology with different CPAs in HPF and NPF. CPA solutions were prepared in DMEM medium. Arrows indicate frost, which formed due to condensation of atmospheric moisture during imaging. Scale bar, 2 mm.

The freezing morphology supported these findings: opaque regions indicated the presence of large ice crystals scattering light (31), while fractures caused by mechanical stress damaged viability and integrity of the sample (32, 33) (Fig. 2). In NPF, increasing penetrable CPA concentrations improved freezing morphology—from complete opacity at 20% EG to partial opacity at 25% EG and complete transparency at 30% EG. Notably, HPF samples maintained a transparent morphology even without penetrable CPAs (Figs. 2 and S3a). However, without dextran, both NPF and HPF samples exhibited severe fracturing (Figs. 2 and S3a). Additionally, pronounced frosting, as indicated by the arrows in Fig. 2, was observed in nondextran samples during imaging, likely due to surface roughness promoting moisture condensation. In contrast, dextran samples had minimal frosting or only a thin mist-like layer, suggesting a smoother surface (Fig. S4).

Previous studies have reported that vitrification on stiff substrates can lead to sample fracturing due to mechanical stress induced by differential thermal expansion (32). Experiments and mathematical models showed that stress magnitude depends on thermal gradients, expansion coefficients, elastic modulus, and sample size (34, 35). In large-volume vitrification studies of organs and tissues, direct immersion into liquid nitrogen leads to severe fracturing. Annealing near the glass transition temperature (*T*_g_) is required to mitigate thermal gradients (8, 33). Based on our findings, we hypothesize that the beneficial effects of dextran may be associated with enhanced fracture toughness. The presence of polymers likely alters the mechanical response of vitrified samples, making them more prone to ductile fracture rather than brittle fracture at cryogenic temperatures (36). To further support our hypothesis, we vitrified 20 mL solution by direct immersion in liquid nitrogen, this normally leads to uneven temperature distribution, resulting in thermal stress and severe sample fracture (33). However, samples containing dextran showed markedly fewer fractures (Fig. S5).

We also tested 20 w/v% sucrose solutions, 40 v/v% EG solution, viscous 40 v/v% glycerol solutions, and viscoelastic 1 w/v% xanthan gum solutions, but all resulted in fracture (Fig. S3). Therefore, we used 20% dextran as the nonpenetrable CPA in subsequent experiments.

### Comparison of HPF and NPF for 2D cell monolayer cryopreservation

**Figure 3:**
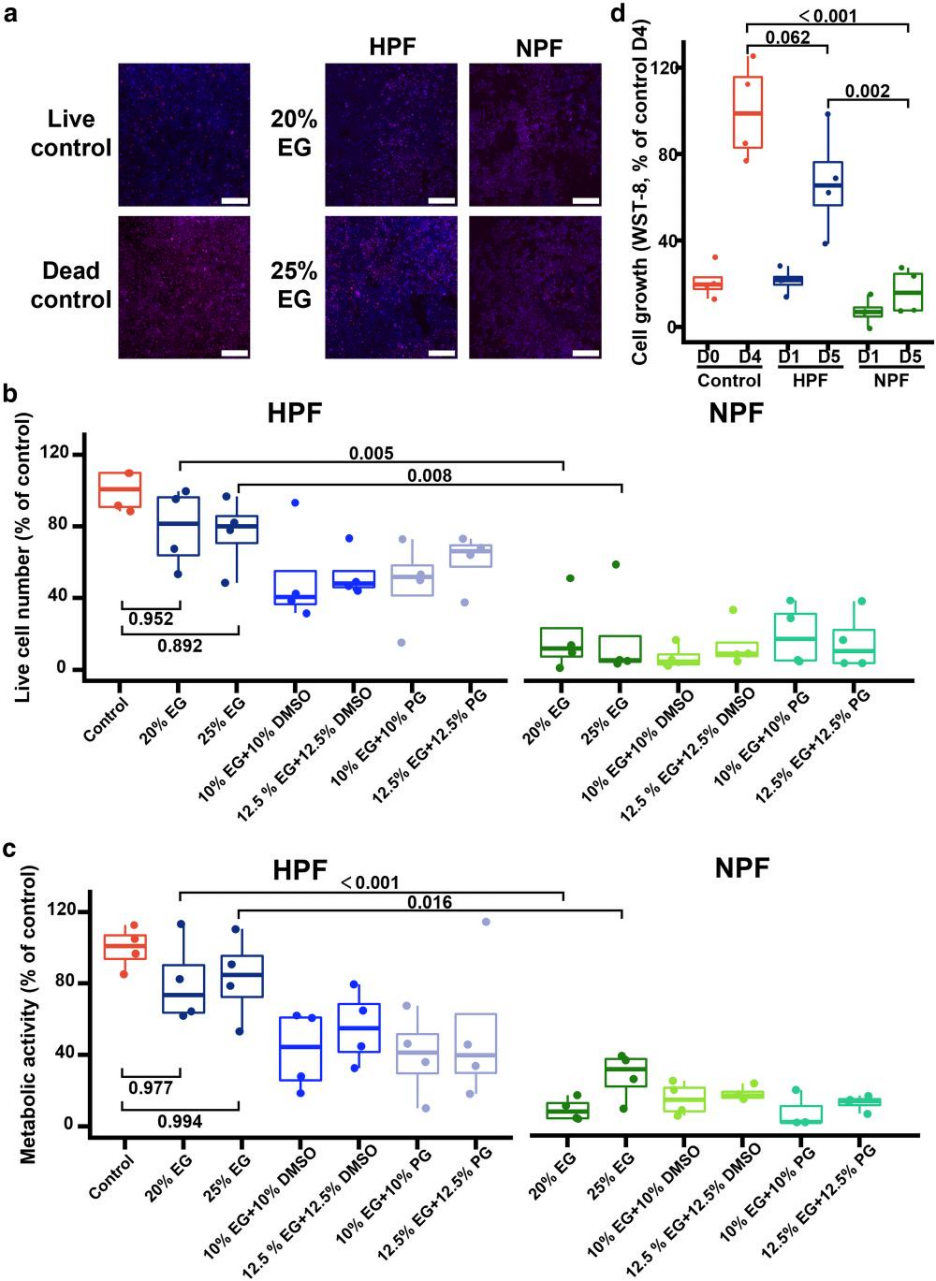
The 2D cell monolayer cryopreservation using HPF and NPF. a) Representative PI/Hoechst merged images of the live control (no treatment), dead control (70% ethanol treatment), and freezing/thawing groups. Dead cells were identified by PI staining, while live cells were defined as Hoechst-positive and PI-negative (shown in magenta and blue, respectively). Scale bars, 500 μm. b) Live cell number (normalized by control group) of the live control and freezing/thawing groups. Six different CPAs (all containing 20% dextran) were used for HPF and NPF. Cell counting was based on PI/Hoechst staining. c) WST-8 absorbance (normalized by control group) of the live control and freezing/thawing groups. To account for potential growth arrest following cryopreservation, for the freezing/thawing groups, WST-8 was measured on day 1 postthawing, while for the live control, it was measured on day 0. d) Cell growth of the live control and freezing/thawing groups. To account for potential growth arrest following cryopreservation, WST-8 assays were performed on day 1 and day 5 postthawing for the freezing/thawing groups and on day 0 and day 4 for the live control. *n* = 4 independent experiments. Statistical analysis was performed using two-way ANOVA with Tukey's post hoc. *P*-values for key pairwise comparisons are shown.

Cell monolayers are susceptible to freezing damage, which can induce cell death and detachment. Previous studies reported that <20% of live cells remained on the substrate after freezing (37, 38). To evaluate the effects of different freezing methods and CPAs on cell monolayers, we performed propidium iodide (PI)/Hoechst staining (Fig. 3a) to quantify live cells (Hoechst^+^ PI^−^), total cells (Hoechst^+^), and cell viability (live cells/total cells) 3 h postthawing. All HPF subgroups exhibited higher live cell numbers than NPF subgroups (Fig. 3b). Consistently, the total cell numbers and viability were higher in HPF (Fig. S6). Within the HPF group, EG groups showed the highest live cell numbers, although the differences were not statistically significant; 20 and 25% EG preserved 79.0 ± 22.2 and 76.4 ± 20.2% of live cells relative to the control, respectively (Fig. 3b).

WST-8 assay, which reflects total cell number and metabolic activity, showed a similar trend to the staining results (Fig. 3c). On postthawing day 1, metabolic activity recovered to 80.5 ± 23.8 and 83.3 ± 24.0% of prefreezing levels in the 20 and 25% EG groups, respectively (Fig. 3c). With 25% EG, HPF cell monolayers exhibited proliferation over 5 days. In contrast, NPF showed minimal proliferation (Fig. 3d). These results demonstrate that HPF is more effective than NPF for preserving cell monolayers. The superior outcomes observed with EG could be attributed to its low toxicity (5, 39), and we, therefore, selected EG as the CPA for subsequent cell spheroid studies.

### Comparison of HPF and NPF for cell spheroid cryopreservation

Next, we examined the cryopreservation of cell spheroids, which have a larger size and 3D structure. Spheroids were generated using 200 μm microwells, as previously described (40). We optimized the CPA loading/unloading protocol. Long CPA incubation time or high CPA concentrations (35% EG) significantly reduced cell viability (Fig. S7), highlighting the need to minimize cytotoxicity.

**Figure 4:**
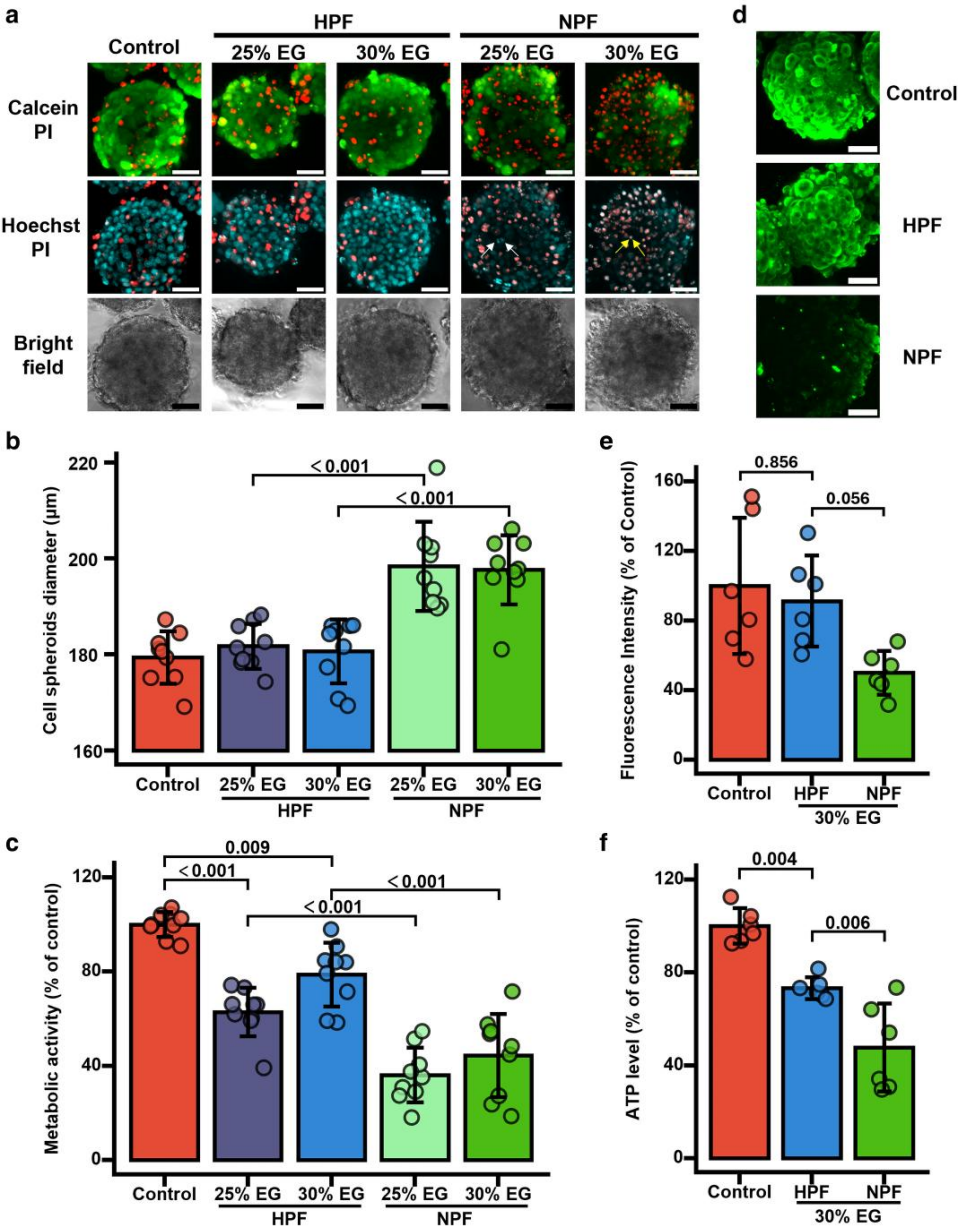
Cell spheroid cryopreservation using HPF and NPF. a) Confocal and bright-field images of cell spheroids from the live control and freezing/thawing groups. Arrows highlight representative cells exhibiting nuclear fragmentation and nuclear condensation. Scale bar, 50 μm. b) Cell spheroid diameter of live control and freezing/thawing groups. Feret's diameters of the cell spheroids in (a) were measured using ImageJ. *n* = 9 independent experiments. c) WST-8 absorbance (normalized by control group) of the live control and freezing/thawing groups. *n* = 9 independent experiments. d) Confocal microscopy image of E-cadherin immunostaining in cell spheroids. For HPF and NPF groups, use 30% EG + 20% dextran as CPA. Scale bar, 50 μm. e) Fluorescence intensity of E-cadherin immunostaining of live control and freezing/thawing groups. Fluorescence intensity was quantified by ImageJ. *n* = 6 independent experiments. f) ATP levels (normalized by control group) of the live control and freezing/thawing groups. *n* = 6 independent experiments. Statistical analysis was performed using two-way ANOVA followed by Tukey's post hoc test for (b, c) and one-way ANOVA followed by Tukey's post hoc test for (e, f). *P*-values for key pairwise comparisons are shown.

Using the optimized protocol, we then assessed the effects of cryopreservation on cell spheroids. Vitrification-induced cell damage was time dependent, becoming apparent ∼3 h postthawing (Fig. S8). To avoid overestimation of cell viability, all evaluations of cell spheroids were conducted at least 5 h postthawing. NPF spheroids exhibited a substantial increase in membrane-damaged cells (PI-stained cells), along with nuclear fragmentation (arrows in NPF 25% EG) and condensation (arrows in NPF 30% EG), which are clear features of apoptosis, as revealed by Calcein-AM/PI/Hoechst staining 5 h postthawing (Fig. 4a). Bright-field imaging showed that NPF spheroids enlarged (Fig. 4a), which was further quantified in Fig. 4b. These spheroids exhibited blurred edges, suggesting loss of tight cell–cell adhesion, which is further supported by the reduced E-cadherin (a cell–cell adhesion molecule) signal (Fig. 4d and e). In contrast, HPF spheroids retained their original size with well-defined boundaries (Fig. 4a and b). Cell viability was markedly improved, particularly in the 30% EG group (Fig. 4a), which also maintained cell–cell adhesion (Fig. 4d and e). In the WST-8 assay, HPF exhibited significantly higher metabolic activity than NPF at both CPA concentrations, with activity further increasing from 62.8 ± 10.3 to 78.8 ± 13.5% of the control as EG concentration rose from 25 to 30% (Fig. 4c). When using 30% EG as CPA, HPF spheroids retained 73.2 ± 4.7% of the control ATP levels, significantly higher than NPF group (Fig. 4f). These findings show that HPF outperforms NPF in preserving cell spheroid viability, metabolic activity, and cell–cell adhesion.

## Discussion

This study provides evidence that HPF enhances cryopreservation outcomes in cultured cells, specifically in monolayers and spheroids—formats that are among the most challenging to preserve. Postfreezing observations revealed that with dextran as a nonpenetrable CPA, HPF samples remained transparent and fracture free even without cytotoxic penetrable CPAs (Fig. 2). A previous study reported that the survival rate of bovine oocytes after HPF was lower compared with the conventional method (41). This may be because the nonpenetrating CPA used in this study was sucrose rather than dextran, which can lead to sample fracture (Fig. S3). In our research, HPF-treated 2D and 3D samples exhibited significantly higher cell viability and metabolic activity after thawing than NPF samples, which developed severe ice crystals under the same CPA conditions (Figs. 3 and 4). Efficient cryopreservation of these cell models by HPF can save time in cell culture, facilitate standardization, and reduce batch-to-batch variability for applications such as drug testing and cell transplantation.

Several vitrification methods with high cooling and warming rates have been developed. For example, the Cryotop (42) and quartz microcapillary (43) methods achieve cooling rates of ∼380 and 1,700 °C/s, respectively. Although the sample volume used in this study is larger, Leica reports that the HPF device employed here can achieve cooling rates of 12,000 to 25,000 °C/s. It should be noted that the measured cooling rate depends on the measurement location, as the cooling rate at the sample surface is much higher than the sample interior. Our results demonstrate that HPF outperforms NPF, suggesting that, for a given sample volume and warming protocol, increasing the cooling rate and pressure during vitrification may further reduce the required CPA concentration.

Current research primarily focuses on optimizing the thawing process (7, 16, 44, 45), as the warming rate required to avoid recrystallization is typically 100–1,000 times higher than the cooling rate required to avoid ice crystallization (46–48). However, the impact of freezing conditions on subsequent recrystallization is overlooked. Since perfect vitrification remains challenging, ice nucleus within amorphous ice trigger recrystallization during thawing. Samples with low ice nucleus density can resist recrystallization at lower warming rates (48–50). Additionally, several studies have shown that freezing at 210 MPa favors the formation of high-density amorphous (HDA) ice rather than low-density amorphous (LDA) ice (51–55). HDA's molecular structure is less compatible with crystalline ice than LDA, potentially functioning as a natural CPA (56). When the temperature rises, HDA first transitions into LDA, releasing a significant amount of latent heat (57), which may increase the warming rate internally. Although further experimental verification is needed, we propose that HPF enhances cryopreservation via two potential mechanisms: reduced ice nucleus density and HDA formation.

CPA selection for HPF relies on empirical knowledge rather than systematic optimization (22). In cryofixation, commonly used CPAs for HPF include dextran, bovine serum albumin (BSA), Ficoll, agarose, and polyvinylpyrrolidone (22). In cryopreservation, heterogeneity in thermal expansion coefficients often leads to fracture, resulting in compromised sample viability or structural integrity (33, 58, 59). We propose that polymeric additives like dextran may shift fracture behavior toward ductile fracture, thereby enhancing resistance against mechanical stress (36). In addition, although clear differences in fracture behavior between HPF and NPF samples are difficult to distinguish in this study, high pressure is known to change the thermal expansion properties of materials, and its effects require further investigation. Although 40 v/v% viscous glycerol and 1 w/v% viscoelastic xanthan gum did not reduce fracture formation, we do not exclude the possibility that viscosity or viscoelasticity may help attenuate pressure waves during rapid pressurization (60, 61), thereby reducing local stress concentrations and mitigating barotoxicity. Besides, given the time limitation of CPA loading to prevent cytotoxicity, the reduced tendency for bubble formation in viscous and viscoelastic solutions was an advantage, since manually removing microbubbles in the sample before HPF was crucial. HPF is a highly complex process involving simultaneous pressure increase, temperature decrease, and glass transition. While our study highlights the value of cross-disciplinary insights, further experimental and modeling studies are required to better characterize HPF.

Additionally, the pressure tolerance of different biological samples requires further investigation, considering variations in their biological composition, structural complexity, and scale. A review by Consiglio et al. (23) summarized studies on the pressure tolerance of various biological samples. While certain samples exhibit damage at pressures above ∼40 MPa, the reported pressure exposure durations are typically in the order of hours or even days. In contrast, some literature suggests that certain biological samples can tolerate 200 MPa for short durations (within 10 ms in this study) (23, 62, 63).

Our results highlight the need for designing HPF devices specifically for cryopreservation. Unlike the HPF devices for cryofixation—which detaches the sample carrier after freezing for microscopy—devices for cryopreservation demand a sealed chamber throughout both freezing and thawing. This would not only help sustain high pressure under isochoric conditions during thawing (56, 64, 65) but also reduce heterogeneous nucleation at the water–air interface (66), thereby limiting recrystallization. Additionally, a sealed chamber also prevents direct sample–liquid nitrogen contact, reducing the risk of contamination, which is an inherent challenge in cryopreservation (67, 68). Moreover, scaling up is essential for application to larger biological samples, such as tissues and organs. Interestingly, large-scale high-pressure devices are already well established in the food industry. High-pressure processing (HPP) and pressure shift freezing are widely used for food sterilization and storage (69). Manufacturers including Hiperbaric, Avure, and Kobelco have developed industrial-scale HPP devices (100–600 MPa) exceeding 500 L, as well as laboratory-scale devices ranging from 0.3 to 10 L (70). Integrating sealed high-pressure chambers with rapid cooling systems, such as multiple liquid nitrogen jets, could expand the application of HPF cryopreservation.

In summary, we conducted a proof-of-concept study on applying HPF for cryopreservation. This study provides the first empirical validation of HPF as a viable and potent cryopreservation strategy, suppressing ice crystallization and maintaining high sample viability at lower CPA concentrations. Notably, HPF samples exhibited a transparent, fracture-free morphology even without penetrable CPAs. Coupling HPF with advanced warming techniques such as joule warming (7) or nanowarming (6, 8, 16) may enable vitrification with low or even CPA-free conditions.

## Materials and methods

### Cell culture

HepG2 cells were cultured in Dulbecco's Modified Eagle Medium (DMEM) supplemented with 10% fetal bovine serum (Wako) and 1% penicillin–streptomycin (Wako) at 37 °C in a 5% CO_2_ incubator. For cell monolayer experiments, cells were seeded on the sample carrier at a density of 1.8 × 10^4^ cells/cm^2^ for general experiments and 6.4 × 10^3^ cells/cm^2^ for cell growth assessment. Cells were cultured for 3 days before further experiments.

For cell spheroid experiments, HepG2 cells were seeded into PDMS microwells (200 μm in diameter) pretreated with Pluronic F-127 (40). The cells were cultured for 4 days to allow spheroid formation before further experiments.

### Freezing and morphology assessment

For freezing, copper flat specimen *ϕ* 6 mm carriers with gold-plated surfaces (Leica Microsystems) commonly used in HPF were used to hold samples. The flat side of the type B carrier was used as the cover and the type A carrier (indentations on both sides, 100 and 200 μm) were used as bottom parts. Before freezing, the carriers were coated with a 1% lecithin solution to facilitate the separation of the cover and bottom parts after freezing. For morphology assessment and cell spheroid cryopreservation, both the cover and bottom were coated. For cell monolayer cryopreservation, only the cover was coated. Each sample was loaded onto the carrier, and the volume of the CPA solution was adjusted to form a slightly convex surface. Microbubbles present in the sample were carefully removed under microscopic observation using a pipette. HPF was conducted using a Leica EM ICE high-pressure freezer, which can complete pressurization and vitrification within 10 ms. NPF was performed by dropping each sample from a height of at least 25 cm into liquid nitrogen. The high density of the sample carriers enabled rapid immersion into liquid nitrogen. High thermal conductivity and rapid immersion contributed to rapid heat transfer (30, 43). Before thawing, each sample was placed on a metal surface precooled with liquid nitrogen for 3 min to remove residual liquid nitrogen surrounding the sample. During this process, the metal was kept immersed in liquid nitrogen, with the liquid level maintained slightly below the surface of the metal. In this case, the metal surface maintained a temperature below −180 °C for at least 10 min. For morphology assessment, imaging was performed using a stereomicroscope (SZX7, DP27 digital CCD camera, Olympus).

### 2D cell monolayer cryopreservation

Six final CPA solutions were prepared in DMEM, containing 20 v/v% or 25 v/v% EG, EG/DMSO (1:1), or EG/PG (1:1), and all six solutions also contained 20 w/v% dextran (40 kDa). For CPA loading, samples were first incubated in 7% EG, EG/DMSO, or EG/PG at 4 °C for 3 min, followed by immersion in the corresponding final CPA solution at 4 °C. The liquid volume in the carrier was adjusted to 2 µL. Samples exposed to the final CPA for ∼1.5 min before freezing. Cryopreserved samples were placed in precooled cryotubes filled with liquid nitrogen and stored in a cryogenic storage dewar. The presence of liquid nitrogen inside the cryotube was monitored to ensure temperature stability.

For thawing, after removing liquid nitrogen around the samples (Fig. 1), the sample was then plunged into a 37 °C 1 M sucrose solution for 30 s, followed by transferring to room temperature 0.5 M sucrose for 3 min to unload CPA. Samples were subsequently washed in culture medium for 3 min before being transferred to standard cell culture conditions for recovery.

### Cell spheroid cryopreservation

For CPA loading, cell spheroids were sequentially incubated at 4 °C in 5% EG for 3 min, 15% EG for 3 min, and in the final CPA solution (25 or 30% EG with 20% dextran) for 2 min. Add 4 µL of the sample to the carrier.

For thawing, the sample was plunged into a 37 °C solution containing either 13% EG + 0.15 M sucrose (for 25% EG) or 16% EG + 0.15 M sucrose (for 30% EG). After 1 min, a 4 °C equal volume of 0.3 M sucrose was added, and the sample was placed on ice. After 4 min, the solution was further diluted 1:1 with 4 °C 0.3 M sucrose and incubated for another 4 min. The spheroids were then washed in room-temperature culture medium, collected, and transferred to a low-adhesion 96-well plate for further culture under standard conditions.

### Fluorescent staining and quantification

Cell monolayers were stained with Hoechst 33342 (2 μg/mL) and PI (0.1 μg/mL) for 15 min at room temperature, imaged using a stereo fluorescence microscope (Leica MZ FL b, Leica Microsystems). A 2.5 mm × 2.5 mm area at the center of the image was cropped, and the number of stained cells was quantified using ImageJ. The results of live cell number and total cell number were normalized to the control group and expressed as a % of control.

Cell spheroids were stained with Calcein-AM (10 μg/mL), Hoechst 33342 (25 μg/mL) and PI (15 μg/mL) for 2 min at room temperature and imaged using an Olympus Fluoview 3000 inverted confocal microscope.

### WST-8 assay

Metabolic activity was assessed 24 h postthawing using the WST-8 assay kit (Cell Counting Kit-8, Dojindo). For cell monolayers, WST-8 assays were performed in 48-well plates, with each well containing 200 μL of culture medium and 20 μL of WST-8 reagent. After 2 h of incubation at 37 °C, absorbance at 450 and 630 nm (reference wavelength) was measured using a microplate reader.

For cell spheroids, WST-8 assays were performed in 96-well plates, with 100 μL of culture medium and 10 μL of WST-8 reagent per well. Incubate at 37 °C for 4 h. The measured values were normalized to the number of spheroids per well (≥15 spheroids per well) to account for metabolic activity per spheroid. All results were normalized to the control group and expressed as % of control.

### ATP measurement

ATP measurement was performed on cell spheroids 24 h postthawing using the CellTiter-Glo 3D Assay kit (Promega). Briefly, 100 μL of room-temperature reagent was added to 100 μL of the sample in a 96-well plate. The plate was then sealed, covered with aluminum foil, and placed on a plate shaker at 100 rpm for 5 min at room temperature. After shaking, the plate was incubated at room temperature for 25 min to stabilize the luminescent signal. Luminescence was measured using a multilabel plate reader. The obtained values were normalized to the number of cell spheroids per well (≥10 spheroids per well).

### Immunofluorescence analysis

Immunofluorescence staining was performed on cell spheroids at 5 h postthawing. Spheroids were fixed with 4% paraformaldehyde for 25 min and blocked in phosphate-buffered saline (PBS) containing 3% BSA and 0.2% Tween-20 for 1 h at room temperature. Samples were then incubated with a primary antibody (3 μg/mL) diluted in PBS containing 3% BSA at 4 °C overnight, followed by an Alexa Fluor 488-conjugated secondary antibody (1 μg/mL) in PBS with 3% BSA for 2 h at room temperature in the dark. Cell nuclei were counterstained with Hoechst 33342 (25 μg/mL) for 2 min. Images were acquired using an Olympus Fluoview 3000 inverted confocal microscope.

### Statistical analysis

Data are presented as mean ± SD, with sample sizes (*n*) indicated in the figure legends. Statistical analyses were performed using R. One-way ANOVA or two-way ANOVA was conducted as appropriate, followed by Tukey's post hoc test for multiple comparisons. *P*-values are directly reported in the figures. The *P*-values of <0.05 were considered statistically significant.

## Supplementary Material

pgag065_Supplementary_Data

## Data Availability

Source data are available for Figs. 3, 4, S3, S6, and S7 in the source data file. Source data are provided with this paper.

## References

[1] Wowk B . 2010. Thermodynamic aspects of vitrification. Cryobiology. 60:11–22. 19538955 10.1016/j.cryobiol.2009.05.007

[2] Mazur P, Leibo SP, Chu EHY. 1972. 2-factor hypothesis of freezing injury-evidence from Chinese-hamaster tissue-culture cells. Exp Cell Res. 71:345–355. 5045639 10.1016/0014-4827(72)90303-5

[3] Behl S, et al 2023. Vitrification versus slow freezing of human ovarian tissue: a systematic review and meta-analysis of histological outcomes. J Assist Reprod Genet. 40:455–464. 36542310 10.1007/s10815-022-02692-w PMC10033773

[4] Wang J, Li R. 2024. Effects, methods and limits of the cryopreservation on mesenchymal stem cells. Stem Cell Res Ther. 15:337. 39343920 10.1186/s13287-024-03954-3 PMC11441116

[5] Zhan L, et al 2022. Pancreatic islet cryopreservation by vitrification achieves high viability, function, recovery and clinical scalability for transplantation. Nat Med. 28:798–808. 35288694 10.1038/s41591-022-01718-1 PMC9018423

[6] Ito A, et al 2020. Magnetic heating of nanoparticles as a scalable cryopreservation technology for human induced pluripotent stem cells. Sci Rep. 10:13605. 32788637 10.1038/s41598-020-70707-6 PMC7423927

[7] Zhan L, et al 2022. Rapid joule heating improves vitrification based cryopreservation. Nat Commun. 13:6017. 36224179 10.1038/s41467-022-33546-9 PMC9556611

[8] Han Z, et al 2023. Vitrification and nanowarming enable long-term organ cryopreservation and life-sustaining kidney transplantation in a rat model. Nat Commun. 14:3407. 37296144 10.1038/s41467-023-38824-8 PMC10256770

[9] Zhan L, et al 2021. Conduction cooling and plasmonic heating dramatically increase droplet vitrification volumes for cell cryopreservation. Adv Sci. 8:2004605. 10.1002/advs.202004605 PMC8188207 34141523

[10] Wowk B, et al 2025. 55 MHz constant field dielectric warming of kidneys and ovaries cryopreserved by vitrification. Cryobiology. 119:105257. 40345110 10.1016/j.cryobiol.2025.105257 PMC12161498

[11] Lee C, et al 2022. Peptide-DNA origami as a cryoprotectant for cell preservation. Sci Adv. 8:eadd0185. 36306364 10.1126/sciadv.add0185 PMC9616499

[12] Fahy GM . 2010. Cryoprotectant toxicity neutralization. Cryobiology. 60:S45–S53. 19501081 10.1016/j.cryobiol.2009.05.005

[13] Deller RC, Vatish M, Mitchell DA, Gibson MI. 2014. Synthetic polymers enable non-vitreous cellular cryopreservation by reducing ice crystal growth during thawing. Nat Commun. 5:3244. 24488146 10.1038/ncomms4244

[14] Matsumura K, Hyon S-H. 2009. Polyampholytes as low toxic efficient cryoprotective agents with antifreeze protein properties. Biomaterials. 30:4842–4849. 19515417 10.1016/j.biomaterials.2009.05.025

[15] Huang H, et al 2015. Alginate hydrogel microencapsulation inhibits devitrification and enables large-volume low-CPA cell vitrification. Adv Funct Mater. 25:6839–6850. 10.1002/adfm.201503047 PMC4667367 26640426

[16] Tian C, et al 2022. Microencapsulation and nanowarming enables vitrification cryopreservation of mouse preantral follicles. Nat Commun. 13:7515. 36522314 10.1038/s41467-022-34549-2 PMC9755531

[17] Koop T, Luo B, Tsias A, Peter T. 2000. Water activity as the determinant for homogeneous ice nucleation in aqueous solutions. Nature. 406:611–614. 10949298 10.1038/35020537

[18] Espinosa JR, et al 2017. Role of salt, pressure, and water activity on homogeneous ice nucleation. J Phys Chem Lett. 8:4486–4491. 28876070 10.1021/acs.jpclett.7b01551

[19] Kanno H, Speedy RJ, Angell CA. 1975. Supercooling of water to-92 C under pressure. Science. 189:880–881. 17812529 10.1126/science.189.4206.880

[20] Studer D, Graber W, Al-Amoudi A, Eggli P. 2001. A new approach for cryofixation by high-pressure freezing. J Microsc. 203:285–294. 11555146 10.1046/j.1365-2818.2001.00919.x

[21] Studer D, Humbel BM, Chiquet M. 2008. Electron microscopy of high pressure frozen samples: bridging the gap between cellular ultrastructure and atomic resolution. Histochem Cell Biol. 130:877–889. 18795316 10.1007/s00418-008-0500-1

[22] McDonald KL, Morphew M, Verkade P, Muller-Reichert T. 2007. Recent advances in high-pressure freezing: equipment- and specimen-loading methods. Methods Mol Biol. 369:143–173. 17656750 10.1007/978-1-59745-294-6_8

[23] Consiglio AN, Rubinsky B, Powell-Palm MJ. 2024. A review of the physical principles of isochoric cryopreservation. Annu Rev Heat Transfer. 27:93–164.

[24] Preciado JA, Rubinsky B. 2010. Isochoric preservation: a novel characterization method. Cryobiology. 60:23–29. 19559692 10.1016/j.cryobiol.2009.06.010

[25] Powell-Palm MJ, et al 2023. Cryopreservation and revival of Hawaiian stony corals using isochoric vitrification. Nat Commun. 14:4859. 37612315 10.1038/s41467-023-40500-w PMC10447501

[26] Solanki PK, Rabin Y. 2023. Is isochoric vitrification feasible? Cryobiology. 111:9–15. 36948380 10.1016/j.cryobiol.2023.03.007

[27] Ali A, et al 2024. Experimental observation of cavity-free ice-free isochoric vitrification via combined pressure measurements and photon counting x-ray computed tomography. Cryobiology. 116:104935. 38936595 10.1016/j.cryobiol.2024.104935

[28] Fahy G, Macfarlane D, Angell C. 1983. Vitrification as an approach to cryopreservation. Cryobiology. 20:699–699. 10.1016/0011-2240(84)90079-8 6467964

[29] Karow AM, Liu WP, Humphries AL. 1970. Survival of dog kidneys subjected to high pressures: necrosis of kidneys after freezing. Cryobiology. 7:122–128. 5498348 10.1016/0011-2240(70)90007-6

[30] Guo Z, et al 2024. Conduction-dominated cryomesh for organism vitrification. Adv Sci. 11:e2303317. 10.1002/advs.202303317 PMC10797434 38018294

[31] Forsyth M, Macfarlane D. 1986. Recrystallization revisited. Cryo Letters. 7:367–378.

[32] Rabin Y, Steif PS, Hess KC, Jimenez-Rios JL, Palastro MC. 2006. Fracture formation in vitrified thin films of cryoprotectants. Cryobiology. 53:75–95. 16784737 10.1016/j.cryobiol.2006.03.013 PMC2190754

[33] Manuchehrabadi N, et al 2017. Improved tissue cryopreservation using inductive heating of magnetic nanoparticles. Sci Transl Med. 9:eaah4586. 28251904 10.1126/scitranslmed.aah4586 PMC5470364

[34] Steif PS, Palastro MC, Rabin Y. 2007. The effect of temperature gradients on stress development during cryopreservation via vitrification. Cell Preserv Technol. 5:104–115. 18185851 10.1089/cpt.2007.9994 PMC2180391

[35] Rabin Y . 2024. Thermomechanics modeling and visualization of physical effects to improve cryopreservation by vitrification. Annu Rev Heat Transfer. 27:245–282.

[36] Wondraczek L, et al 2022. Advancing the mechanical performance of glasses: perspectives and challenges. Adv Mater. 34:e2109029. 34870862 10.1002/adma.202109029

[37] Pless-Petig G, Knoop S, Rauen U. 2018. Serum- and albumin-free cryopreservation of endothelial monolayers with a new solution. Organogenesis. 14:107–121. 30081735 10.1080/15476278.2018.1501136 PMC6150062

[38] Stokich B, et al 2014. Cryopreservation of hepatocyte (HepG2) cell monolayers: impact of trehalose. Cryobiology. 69:281–290. 25127872 10.1016/j.cryobiol.2014.08.001

[39] Wolkers WF, Oldenhof H, editors. Cryopreservation and freeze-drying protocols. Vol. 2180, 4th ed. Humana Press, New York, NY, 2021, p. 742.

[40] Shinohara M, et al 2014. Combination of microwell structures and direct oxygenation enables efficient and size-regulated aggregate formation of an insulin-secreting pancreatic β-cell line. Biotechnol Prog. 30:178–187. 24265060 10.1002/btpr.1837

[41] Reader KL, et al 2022. High pressure frozen oocytes have improved ultrastructure but reduced cleavage rates compared to conventionally fixed or vitrified oocytes. Reprod Fertil Dev. 34:1135–1144. 36318972 10.1071/RD22118

[42] Kuwayama M, Vajta G, Kato O, Leibo SP. 2005. Highly efficient vitrification method for cryopreservation of human oocytes. Reprod Biomed Online. 11:300–308. 16176668 10.1016/s1472-6483(10)60837-1

[43] He X, Park EYH, Fowler A, Yarmush ML, Toner M. 2008. Vitrification by ultra-fast cooling at a low concentration of cryoprotectants in a quartz micro-capillary: a study using murine embryonic stem cells. Cryobiology. 56:223–232. 18462712 10.1016/j.cryobiol.2008.03.005 PMC2728604

[44] Seki S, Mazur P. 2009. The dominance of warming rate over cooling rate in the survival of mouse oocytes subjected to a vitrification procedure. Cryobiology. 59:75–82. 19427303 10.1016/j.cryobiol.2009.04.012 PMC2729265

[45] Jin B, Mazur P. 2015. High survival of mouse oocytes/embryos after vitrification without permeating cryoprotectants followed by ultra-rapid warming with an IR laser pulse. Sci Rep. 5:9271. 25786677 10.1038/srep09271 PMC4365397

[46] Han Z, Bischof J. 2020. Critical cooling and warming rates as a function of CPA concentration. Cryo Letters. 41:185–193. 33988646 PMC10186587

[47] Kangas J, et al 2022. Ultra-rapid laser calorimetry for the assessment of crystallization in low-concentration cryoprotectants. J Heat Transfer. 144:031207. 35833150 10.1115/1.4052568 PMC8823201

[48] Hopkins JB, Badeau R, Warkentin M, Thorne RE. 2012. Effect of common cryoprotectants on critical warming rates and ice formation in aqueous solutions. Cryobiology. 65:169–178. 22728046 10.1016/j.cryobiol.2012.05.010 PMC3500404

[49] Mehl PM . 1993. Nucleation and crystal growth in a vitrification solution tested for organ cryopreservation by vitrification. Cryobiology. 30:509–518. 11987991 10.1006/cryo.1993.1051

[50] Abdelhady AW, et al 2024. Ice formation and its elimination in cryopreservation of oocytes. Sci Rep. 14:18809. 39138273 10.1038/s41598-024-69528-8 PMC11322307

[51] Lepault J, Bigot D, Studer D, Erk I. 1997. Freezing of aqueous specimens: an X-ray diffraction study. J Microsc. 187:158–166.

[52] Al-Amoudi A, Dubochet J, Studer D. 2002. Amorphous solid water produced by cryosectioning of crystalline ice at 113 K. J Microsc. 207:146–153. 12180960 10.1046/j.1365-2818.2002.01051.x

[53] Yakovlev S, Downing KH. 2011. Freezing in sealed capillaries for preparation of frozen hydrated sections. J Microsc. 244:235–247. 22077543 10.1111/j.1365-2818.2011.03575.x PMC4199587

[54] Kim CU, Chen Y-F, Tate MW, Gruner SM. 2008. Pressure-induced high-density amorphous ice in protein crystals. J Appl Crystallogr. 41:1–7.

[55] Richter K . 1994. High-density morphologies of ice in high-pressure frozen biological specimens. Ultramicroscopy. 53:237–249. 8160307 10.1016/0304-3991(94)90037-x

[56] Eltareb A, Lopez GE, Giovambattista N. 2021. The role of high-density and low-density amorphous ice on biomolecules at cryogenic temperatures: a case study with polyalanine. Phys Chem Chem Phys. 23:19402–19414. 34494044 10.1039/d1cp02734d PMC8491127

[57] Handa YP, Mishima O, Whalley E. 1986. High-density amorphous ice. III. Thermal properties. J Chem Phys. 84:2766–2770.

[58] Rutt T, et al 2019. Thermal expansion of substrate may affect adhesion of Chinese hamster fibroblasts to surfaces during freezing. Cryobiology. 86:134–139. 30312591 10.1016/j.cryobiol.2018.10.006

[59] Tomás RMF, Dallman R, Congdon TR, Gibson MI. 2023. Cryopreservation of assay-ready hepatocyte monolayers by chemically-induced ice nucleation: preservation of hepatic function and hepatotoxicity screening capabilities. Biomater Sci. 11:7639–7654. 37840476 10.1039/d3bm01046e PMC10661096

[60] Liu L, Fan Y, Li W. 2014. Viscoelastic shock wave in ballistic gelatin behind soft body armor. J Mech Behav Biomed Mater. 34:199–207. 24607758 10.1016/j.jmbbm.2014.02.011

[61] Rezapour S, Riasi A. 2021. Impact of fluid viscoelasticity on the pressure wave in laminar fluid hammer in helical tubes-an experimental study. Int Commun Heat Mass Transfer. 125:105356.

[62] Onuchic LF, Lacazvieira F. 1985. Glycerol-induced baroprotection in erythrocyte membranes. Cryobiology. 22:438–445. 3931983 10.1016/0011-2240(85)90155-5

[63] Erk I, Nicolas G, Caroff A, Lepault J. 1998. Electron microscopy of frozen biological objects: a study using cryosectioning and cryosubstitution. J Microsc. 189:236–248. 9588022 10.1046/j.1365-2818.1998.00323.x

[64] Huebinger J, Han H-M, Grabenbauer M. 2016. Reversible cryopreservation of living cells using an electron microscopy cryo-fixation method. PLoS One. 11:e0164270. 27711254 10.1371/journal.pone.0164270 PMC5053471

[65] Kim KH, et al 2020. Experimental observation of the liquid-liquid transition in bulk supercooled water under pressure. Science. 370:978–982. 33214280 10.1126/science.abb9385

[66] Huang H, Yarmush M, Usta OB. 2018. Long-term deep-supercooling of large-volume water and red cell suspensions via surface sealing with immiscible liquids. Nat Commun. 9:3201. 30097570 10.1038/s41467-018-05636-0 PMC6086840

[67] Parmegiani L, et al 2012. A reliable procedure for decontamination before thawing of human specimens cryostored in liquid nitrogen: three washes with sterile liquid nitrogen (SLN2). Fertil Steril. 98:870–875. 22795638 10.1016/j.fertnstert.2012.06.028

[68] Bielanski A, Vajta G. 2009. Risk of contamination of germplasm during cryopreservation and cryobanking in IVF units. Hum Reprod. 24:2457–2467. 19561041 10.1093/humrep/dep117

[69] Rastogi NK, Raghavarao KSMS, Balasubramaniam VM, Niranjan K, Knorr D. 2007. Opportunities and challenges in high pressure processing of foods. Crit Rev Food Sci Nutr. 47:69–112. 17364696 10.1080/10408390600626420

[70] Huang H-W, Wu S-J, Lu J-K, Shyu Y-T, Wang C-Y. 2017. Current status and future trends of high-pressure processing in food industry. Food Control. 72:1–8.

