# Supplementary material for: A proof-of-concept study on high-pressure freezing for cryopreservation
Source: PNAS Nexus. 2026 Mar 20;5(4):pgag065. doi: 10.1093/pnasnexus/pgag065 (PMC13069681; doi:10.1093/pnasnexus/pgag065)
Supplement: pgag065_Supplementary_Data [file pgag065_supplementary_data.zip › PNASNEXUS-PNASNEXUS-2025-00898-TRR-s03.pdf]

Supporting Information for

A proof-of-concept study on high pressure freezing for cryopreservation

Fang Song[1*], Mayuko Sato[2], Yuya Toyama[1], Taiyo Ishikawa[1], Fumiya Tokito[1], Takeshi Katsuda[1], Kiminori Toyooka[2], Yasuyuki Sakai[1], Masaki Nishikawa[1*]

[1] Department of Chemical System Engineering, Graduate school of Engineering, the University of Tokyo, Tokyo, Japan

[2] Center for Sustainable Resource Science, RIKEN, Yokohama, Japan

* Fang Song, Masaki Nishikawa

Email: song-fang1127@g.ecc.u-tokyo.ac.jp; masaki@chemsys.t.u-tokyo.ac.jp

This PDF file includes:

Figures S1 to S8

Legends for Movies S1, S2

Other supporting materials for this manuscript include the following:

Movies S1, S2

Fig S1

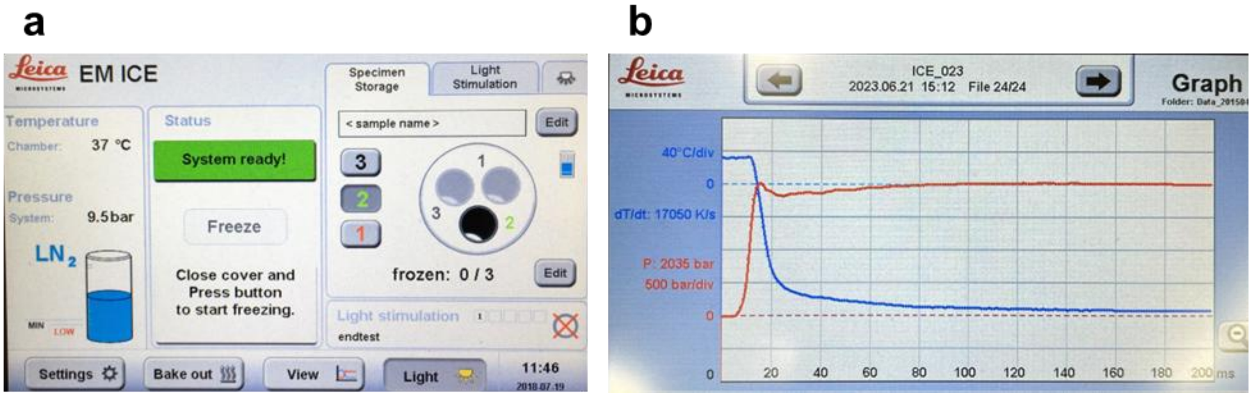

Operation panel of the Leica EM ICE. **a)** One-click operation panel of the Leica EM ICE; after mounting the carrier onto the specimen holder, HPF is initiated by pressing “Freeze.” **b)** Representative temperature–pressure–time profile during an HPF cycle.

Fig. S2.

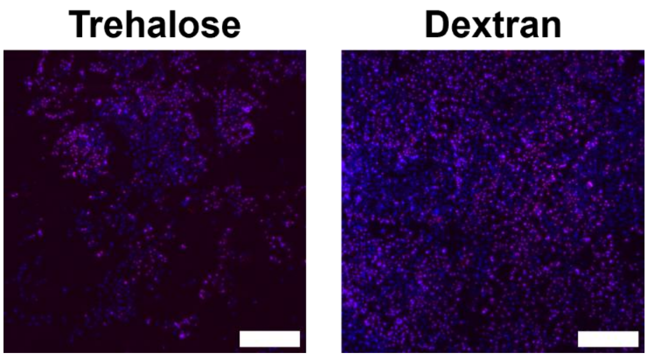

Cell monolayers cryopreservation using trehalose and dextran in HPF. Representative PI/Hoechst merged images of freezing/thawing cell monolayers. Both groups’ CPAs contained 10 v/v% EG and 10 v/v% DMSO, with either 0.5 M trehalose or 20 w/v% dextran. Scale bar, 500 μm.

Fig. S3.

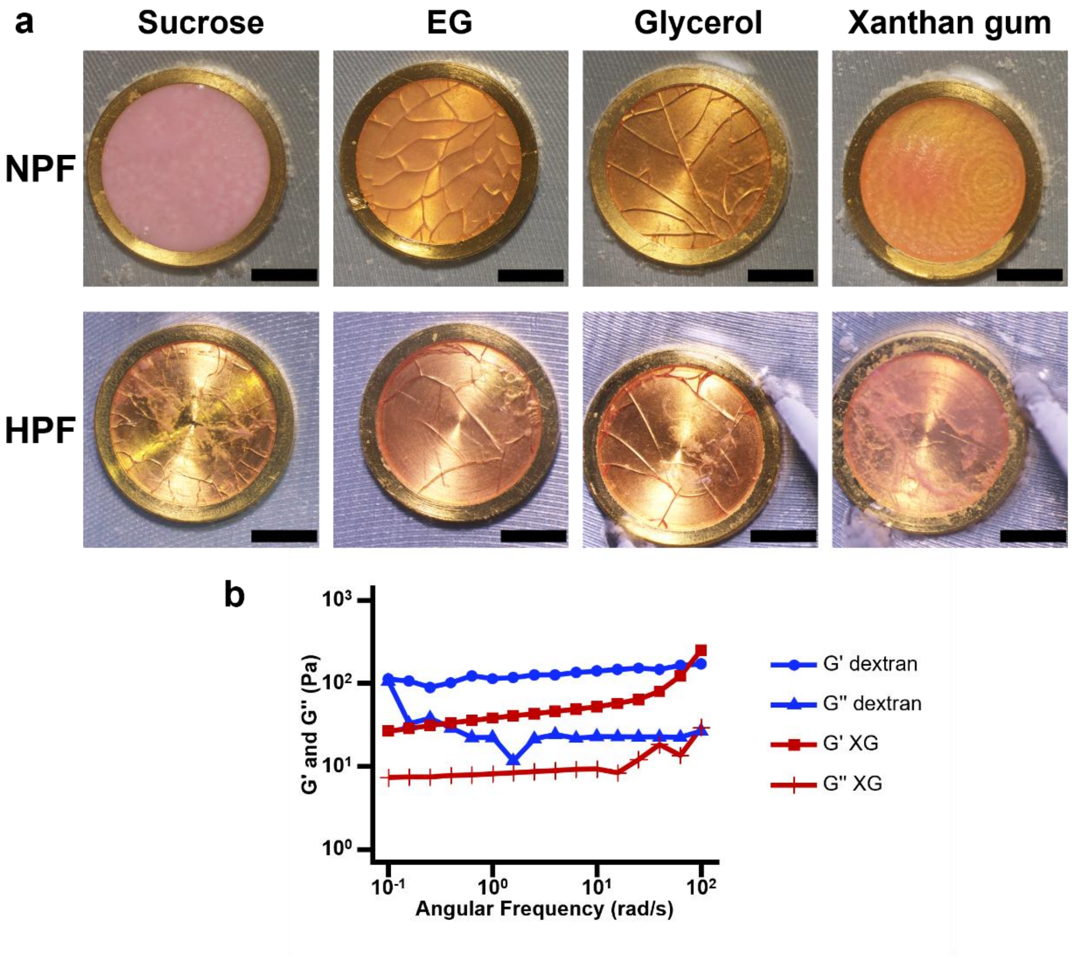

Freezing morphology assessment of different CPAs to investigate the factors for favorable outcomes of dextran. **a)** The freezing morphology of samples. CPA solutions were prepared in DMEM medium, with 20 w/v% sucrose, 40 v/v% EG, 40 v/v% glycerol and 1 w/v% xanthan gum. Scale bar, 2 mm. **b)** Elastic modulus *G'* and loss modulus *G''* as a function of angular frequency for 20 w/v% dextran solutions and 1 w/v% xanthan gum solution. The frequency sweep was measured using an Anton Paar Rheocompass rheometer at a temperature of 25 °C and a shear strain of 0.1%.

Fig S4.

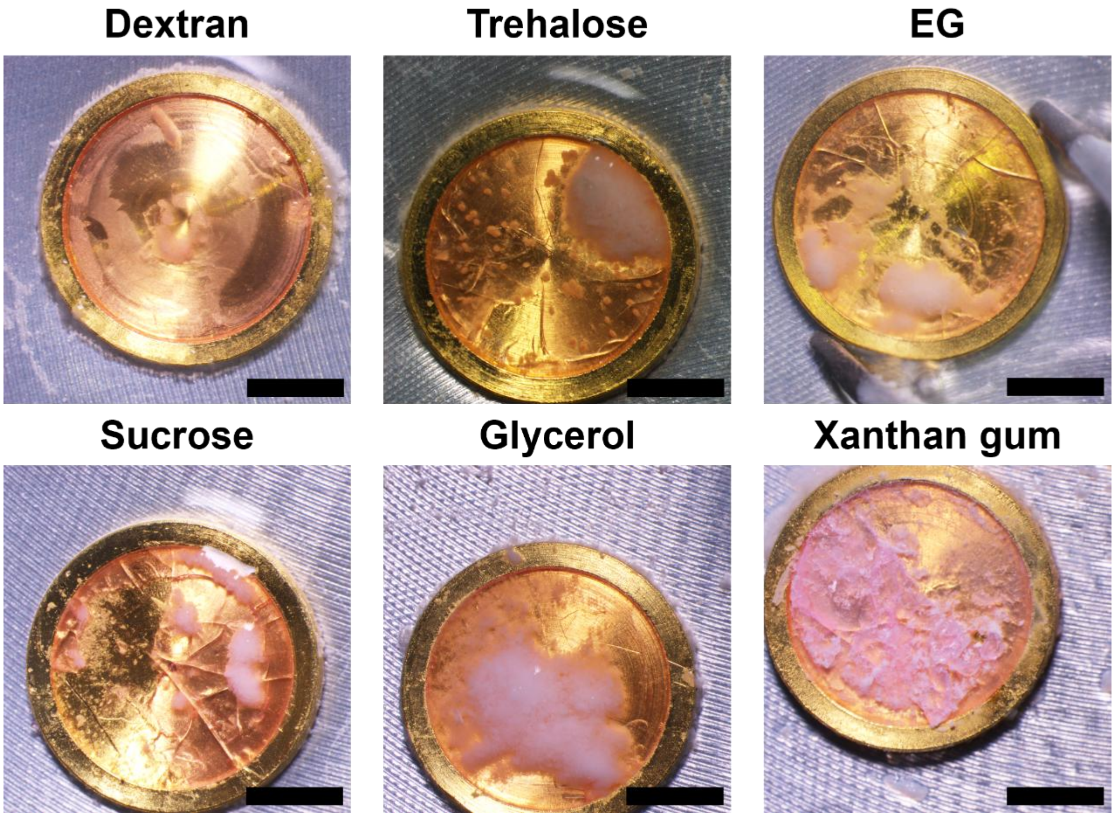

Frost morphology with different CPA during imaging. Images of HPF samples with severe frost formation. Despite our efforts to minimize frost, it occasionally formed instantly when the samples were exposed to air. CPA solutions were prepared in DMEM medium, with 20 w/v% dextran, 20% w/v trehalose, 40 v/v% EG, 20 w/v% sucrose, 40 v/v% glycerol and 1 w/v% xanthan gum. Trehalose sample contained cell spheroids. Scale bar, 2 mm.

Fig S5.

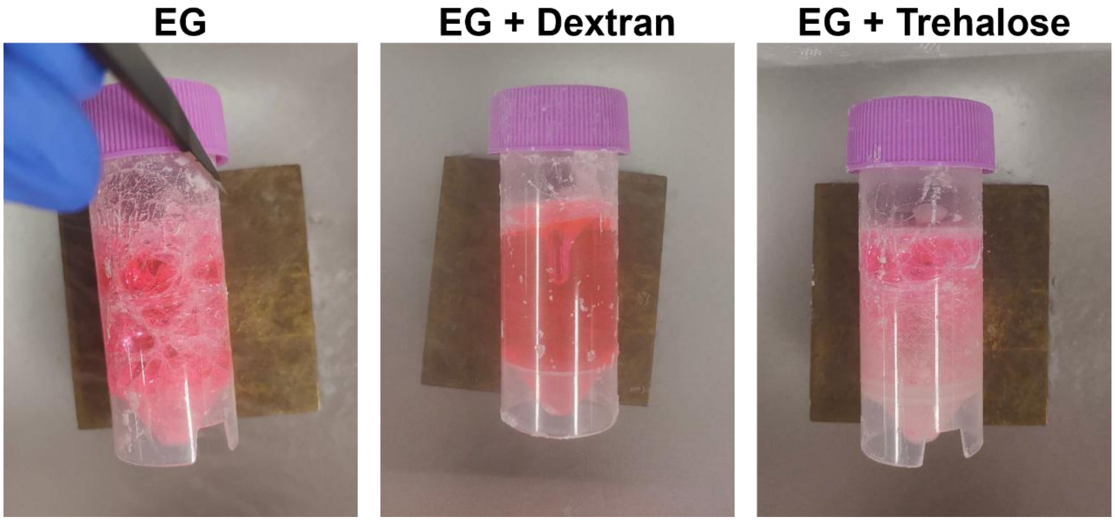

Freezing morphology of 20 mL samples after liquid nitrogen immersion. CPA solutions were prepared in DMEM medium, with 60 v/v% EG, 40 v/v% EG + 25 w/v% dextran and 40 v/v% EG + 25 w/v% trehalose. Samples were placed on a pre-cooled copper block for imaging.

Fig S6.

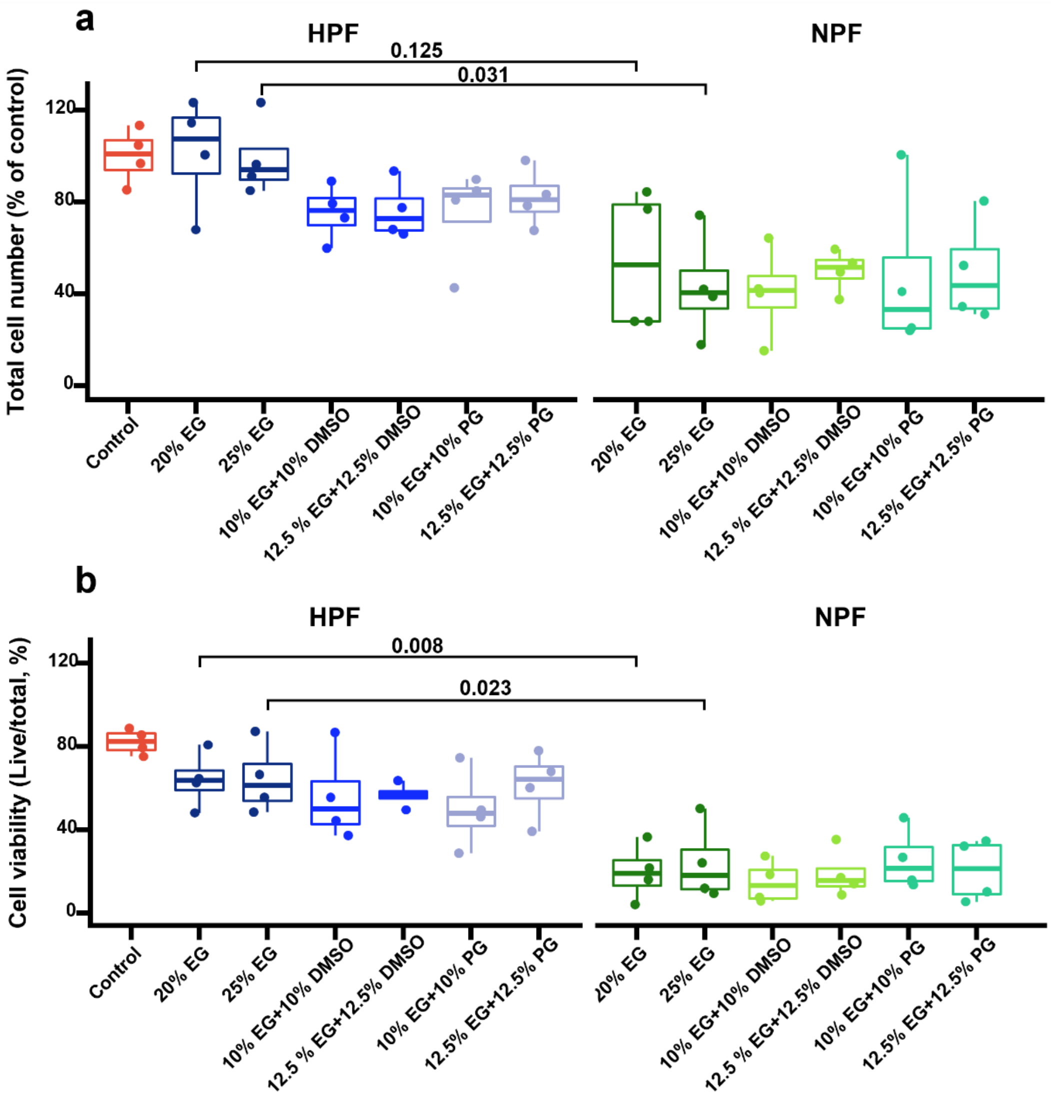

Total cell number **a)** and cell viability **b)** of the live control and freezing/thawing cell monolayers. Cell counting was based on PI/Hoechst staining. n=4 independent experiments. Two-way ANOVA and Tukey's post hoc were used for statistical analysis. P values from informative pairwise comparisons are shown.

Fig S7.

a

Long CPA loading time	Short CPA loading time
7% EG/5 min	5% EG/3 min
20% EG/5 min	15% EG/3 min
25, 30, 35% EG+20% Dextran/3 min	25, 30, 35% EG+20% Dextran/2 min
**Freezing/thawing**	
13, 16, 18% EG+0.15 M sucrose/3 min	13, 16, 18% EG+0.15 M sucrose/1 min
Dilute 2-fold with 0.3 M sucrose/7 min	Dilute 2-fold with 0.3 M sucrose/4 min
Dilute 2-fold with 0.3 M sucrose/7 min	Dilute 2-fold with 0.3 M sucrose/4 min
Culture medium	Culture medium

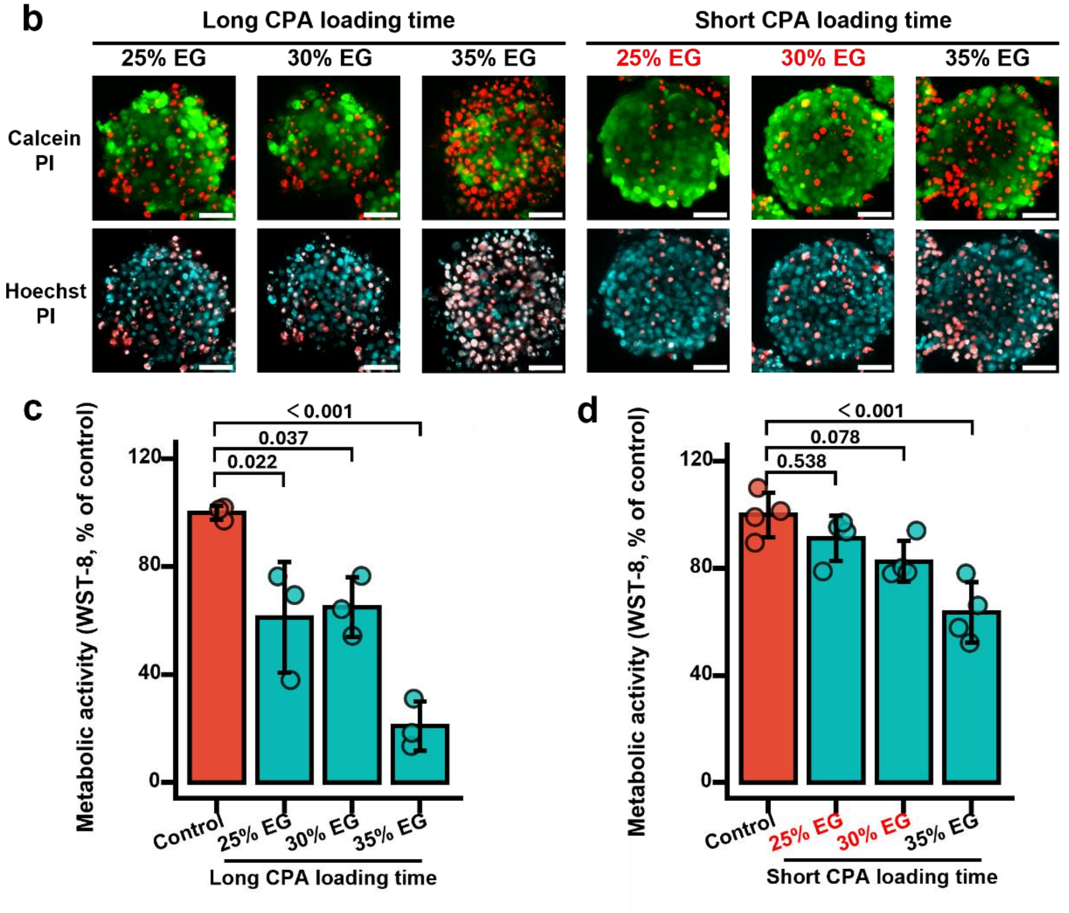

Optimizing CPA loading/unloading protocol for vitrification of cell spheroids. **a)** Two CPA loading/unloading protocols. All steps were performed at 4 °C. **b)** Confocal microscope images of cell spheroids for live control and CPA loading/unloading group. The sample only undergoes CPA loading/unloading but not freezing/thawing. Two loading/unloading protocols and three final concentrations were examined. **c)**, **d)** WST-8 absorbance (normalized by control group) of live control and CPA loading/unloading groups. The red

font highlights the conditions for following vitrification experiments. n=3 for c. n=4 for d. One-way ANOVA and Tukey's post hoc were used for statistical analysis. P values from informative pairwise comparisons are shown.

Fig S8.

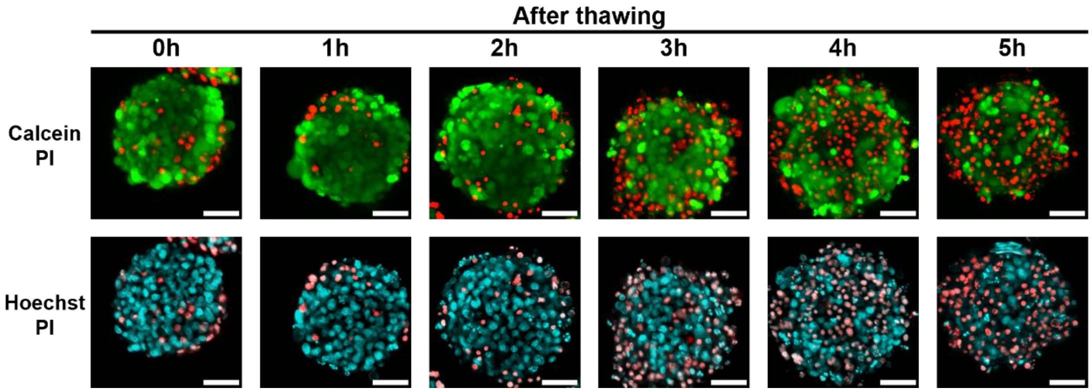

Confocal images of freezing/thawing cell spheroids showing time-dependent cell damage effects of vitrification. Use NPF with 25% EG+20% dextran as CPA. scale bar= 50 μm.

Movie S1 (separate file). Workflow for mounting the carrier onto the specimen holder and performing HPF.

Movie S2 (separate file). Leidenfrost effect on sample plunge during thawing.
